# Local controllability of hot electron and thermal effects enabled by chiral plasmonic nanostructures

**DOI:** 10.1515/nanoph-2021-0780

**Published:** 2022-02-16

**Authors:** Bowen Kang, Tingting Zhang, Lei Yan, Chengxiang Gou, Zihe Jiang, Min Ji, Li Chen, Zhenglong Zhang, Hairong Zheng, Hongxing Xu

**Affiliations:** School of Physics and Information Technology, Shaanxi Normal University, Xi’an, 710062, China; Department of Electrical and Computer Engineering, University of California San Diego, La Jolla, California, USA; School of Physics and Technology, Wuhan University, Wuhan, Hubei, China

**Keywords:** chiral photochemistry, circular dichroism, hot electrons, photothermal effect, plasmonic resonance

## Abstract

The control of hot electron (HE) and thermal effects induced by plasmonic nanostructures has recently attracted considerable attention. When illuminated by light with different circular polarization states, the circular dichroism signal of molecules adsorbed by plasmonic chiral nanostructures can control HE and thermal effects. These effects have the potential to enhance reaction rates and to change selectivity patterns in photothermal catalysis. Here, we propose an aluminum L-shaped chiral nanostructure system in which HE and thermal effects can be controlled in different regions of the nanostructure by changing the chirality of the excitation light. A large difference of 12.75% in the HE effect but a virtually identical thermal effect can be achieved in different regions of the nanostructure by selecting the appropriate probed region, while a large thermal effect difference of 65.67% but a virtually identical HE effect can be achieved in one region of the nanostructure by changing the polarization state of the excitation light. In addition, the HE and thermal chiral selectivity effects of double L-shaped nanostructures are investigated as these structures can be more easily controlled during asymmetric chiral growth and crystallization. This work combined with plasmonic chirality is beneficial for quantifying HE and thermal effects in photochemical reactions and provides theoretical support for designing catalysts and optimizing plasmonic platforms. Additionally, the local controllability of HE and thermal effects plays an essential role in high-resolution photochemical reactions, especially in single-molecule photochemical reactions.

## 1 Introduction

Surface plasmon has recently emerged as a route for driving chemical reactions using hot carrier generation, local heating effects, and optical near-field enhancement [1, 2]. The HE and thermal effects of localized surface plasmon resonance (LSPR) arise from the different processes of surface plasmon decay. Plasmon energy can be nonradiatively dissipated by absorption within a metal nanoparticle and generate energetically hot charge carriers in a plasmonic structure [3, 4]. The hot carriers will redistribute their energy by electron–electron and electron–phonon scattering processes, converting their energy into heat. Eventually, heat is transferred to the surroundings of the metallic structure, causing a thermal effect [5]. Plasmonic HE and thermal effects are promising approaches for the facilitation of photothermal therapy, photodetection, and plasmon-mediated chemical conversions [6], [7], [8]. Unfortunately, accurate measurement of the HE generation rate and the surface temperature at reaction regions remain exceptionally difficulty tasks for current techniques. Quantification of these two effects is paramount for designing catalysts and optimizing plasmonic platforms. Furthermore, traditional photochemistry reactions are usually related to the overall properties of a nanostructure, such as size, shape, and material [9, 10]. To date, an understanding of chiral responses between different regions on one nanostructure, which plays a key role in enantioselective catalysis, has been lacking [11, 12]. For these reasons, it is necessary to locally control HE and thermal effects in plasmonic photochemistry reactions.

Due to the circular dichroism (CD) signal based on the difference in the absorption of left-circularly polarized (LCP) light and right-circularly polarized (RCP) light, chiral nanostructures/molecules have several potential applications in the fields of medicine, biology, and chemistry [13], [14], [15], [16]. Nevertheless, chiral molecules and biomolecules in nature do not generate significant CD signals, which limit their relevant applications. The detected CD signal of chiral molecules can be strongly enhanced using chiral plasmonic nanostructures due to strong interaction with circularly polarized light (CPL) [17, 18]. It should be emphasized that a platform with strong CD signal possesses more robust chiral photothermal responses [19], [20], [21], [22], which is significant in controlling the HE and thermal effects in plasmonic photochemistry. In addition, the material properties of aluminum enable low thermal conductivity and strong plasmon resonances spanning much of the visible region of the spectrum and into the ultraviolet [23, 24]. Combined with its natural abundance, low cost, and amenability, aluminum can be employed as a highly promising catalyst for plasmonic photochemistry [25].

Here, HE and thermal effects are locally controlled by changing the chirality of the excitation light, which is dependent on aluminum L-shaped chiral nanostructures (LCNs). The absorbance spectra and distributions of the HE generation rate and temperature increase on an aluminum LCN are simulated using the finite element method (FEM). The local electric field and absorption of the LCN illuminated with different CPL can vary significantly, leading to non-negligible asymmetry of HE and thermal effects, respectively. The HE effect of the LCN can be regulated by selecting the probed region and the thermal effect of LCN can be controlled by switching the circular polarization state of the excited light. Furthermore, the dynamics of temperature increase of an LCN with CPL excitation are analyzed, which provide value to the study of time-dependent catalytic processes. In addition, the HE and thermal chiral selective effects remain strong for the double-LCN, which is more likely to be manipulated in realistic photochemistry. The theoretical results of this work will help quantify these two effects in photochemical reactions, which is crucial for designing catalysts and optimizing a plasmonic platform. The local controllability of HE and thermal effects on the micro- or nano-scale plays a key role in high-resolution single-molecule photochemical reactions.

## 2 Simulation

### 2.1 Theoretical framework

The differential optical response of a nanostructure can be characterized via CD spectroscopy with CPL, which is one of the most effective methods for measuring chiral asymmetry signal. The optical chiral effect (CD_abs_) is expressed as [13, 14]:

(1) $$CD_{abs}=\sigma_{LCP}-\sigma_{RCP}$$

where *σ*_LCP_ and *σ*_RCP_ are the absorption cross sections of the chiral nanostructure with LCP and RCP excitations, respectively. In the simulation, the absorption cross section is obtained by the integration of ohmic heating within the whole nanostructure (see Supporting information part 2).

In the photocatalytic reaction, heating mechanisms are usually implemented to improve the energy of molecular thermal movement and for realizing molecular activation, thus promoting the breaking of chemical bonds and the production of chemical reactions [26, 27]. Under continuous wave excitation of a plasmonic structure, the local temperature generated by LSPR will increase. Using metallic nanoparticles as nanosources of heat, the temperature increase on the nanostructure is determined by [28, 29]

(2) $$\Delta T\left(r\right)=\frac{Q\left(r\right)}{4\pi \kappa_{s}R_{NP}}=\frac{\sigma I_{0}}{4\pi \kappa_{s}R_{NP}}\text{,}$$

where *Q*(***r***) is the heat power intensity, *σ* is the absorption cross section, *I*_0_ is the incident light flux, *κ*_s_ is the thermal conductivity of the surrounding medium, *R*_NP_ is the radius of the nanoparticle, and ***r*** is the coordinate. The determination of the temperature distribution of the nanostructure is based on the resolution of the heat diffusion equation [19, 20].

(3) $$\rho \left(r\right)c\left(r\right)\frac{\partial }{\partial t}T\left(r\right)=\vec{\nabla }\left[k\left(r\right)\vec{\nabla }T\left(r\right)\right]+Q\left(r\right).$$

In Eq. (3), *ρ*(***r***), *c*(***r***), and *k*(***r***) are the mass density, specific heat, and thermal conductivity, respectively. The local increase of temperature *δT*, is equal to *T*_(***r***)_ − *T*_0_, here *T*_0_ is the initial temperature. Using Eqs. (2) and (3) we can observe that the thermal effect depends on the absorption cross section of the nanoparticle. Plasmon-induced HEs are regarded as a key factor for the catalysis process because the efficiency of a photochemistry reaction depends on the HE generation rate [30], [31], [32]. Based on this observation, the quantum formalism is used to describe the rate of HE generation in the plasmonic nanostructure [33], [34], [35], [36]:

(4) $$Rate_{HEs}=\frac{2}{\pi^{2}}\times \frac{e^{2}E_{\text{F}}^{2}}{\hbar }\frac{\left(\hbar \omega -\Delta E_{b}\right)}{{\left(\hbar \omega \right)}^{4}}\times \iint {\left|E_{nomal}\right|}^{2}ds$$

where ∆*E*_b_ is the energy spacing between the Fermi level and the level of adsorbed molecule, *E*_normal_ is the electric field normal to the metal surface, and the integral is taken over the surface of the nanostructure. It can be observed that the HE effect is dependent on the distribution of the local electric field of the nanostructure.

### 2.2 Computational models

**Figure 1:**
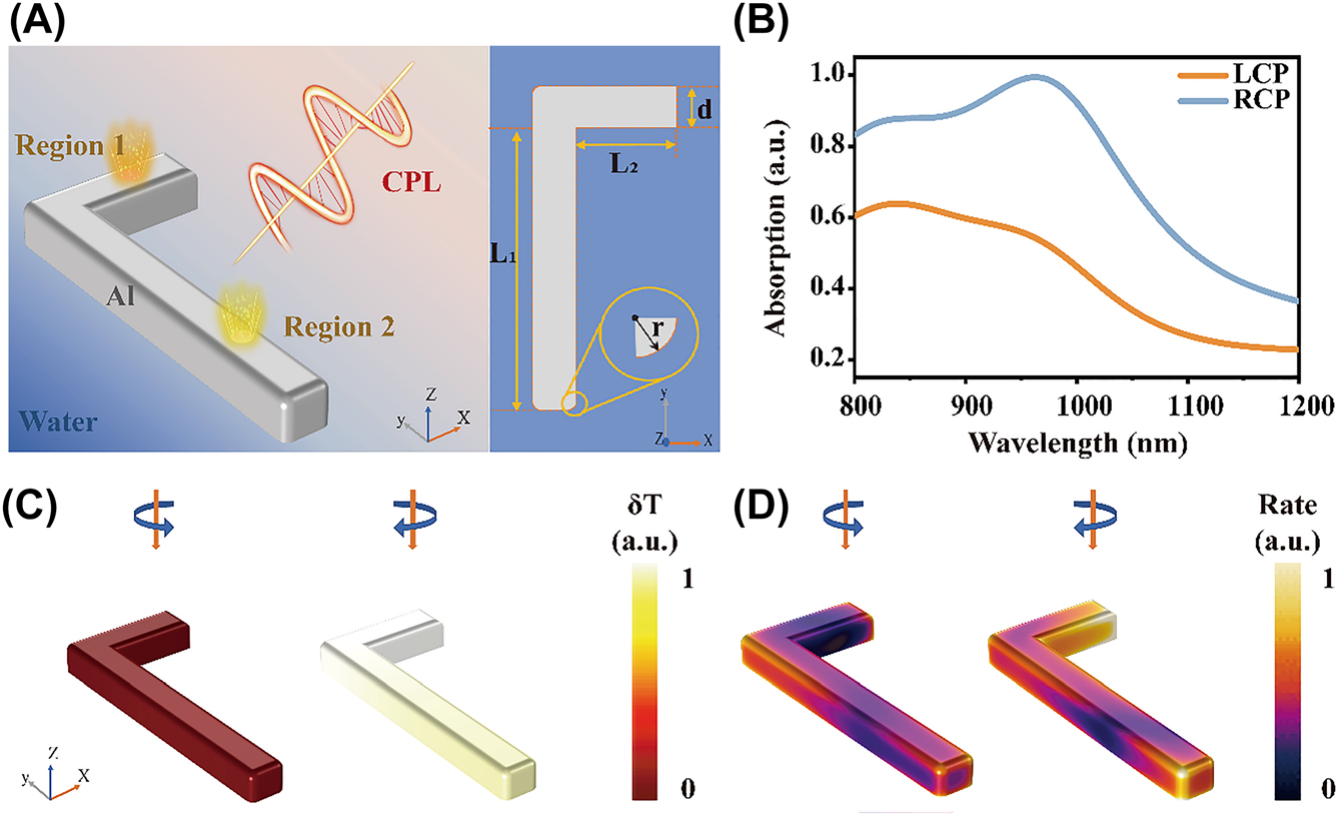
Optical properties of the single LCN of an array. (A) Schematic of the chiral effect and shape parameters of the LCN. (B) Absorbance spectra of the LCN with LCP and RCP excitation. (C) Distributions of normalized temperature increase and (D) HE generation rate of LCN with 980 nm CPL excitation.

To demonstrate the chiral effect, we choose an L-shaped geometry for the nanostructure. This shape is a representative elementary structure for chiral enhancement due to its obvious geometric asymmetry that leads to a strong chiral response [37]. The optical properties of the LCN are simulated using COMSOL Multiphysics software through the FEM. A schematic diagram and the optical response of the LCN with CPL excitation are shown in Figure 1. Details of the model are shown in Figure S1, and the width (*d*), thickness (*h*), and fillet radius (*r*) of the LCN are 50, 40, and 10 nm, respectively. Optical and thermal calculations are performed in a unit cell of the array with periodic boundary conditions applied to the side boundaries. The initial temperature of the nanostructure is set as *T*_0_ = 293.15 K and surrounded by water, in consideration of its application in photocatalysis. The excitation light illuminates the nanostructure from the top boundary in the normal direction. LSPR has many applications which require meeting specific *c* wavelength windows. The most prominent examples are photothermal therapy in biology, matched to the biological window (650–1350 nm) [38]. Thus, we further optimized the size of the structure so that its absorption resonance peak is about 980 nm. The absorption properties of the LCN are investigated in detail to evaluate the chiral response.

The normalized absorption spectra of the LCN with CPL excitation are shown in Figure 1B. The absorption with RCP excitation is much larger than that with LCP excitation, resulting in a strong LCN CD signal. The distribution of the local temperature increase and the HE generation rate of the LCN with CPL excitation are shown in Figure 1C and D, respectively; both HE and thermal effects have a chiral distribution. Due to the higher absorption efficiency, the average temperature increase of the LCN with RCP excitation is higher than that with LCP excitation. The rate of HE generation has been normalized on the LCN, which is beneficial to observe the difference in HE effects of different regions. According to the previous experimental reports, the HEs can be detected directly or indirectly [7, 39]. Meanwhile, the distribution of the HE generation rate also exhibits obvious differences in different regions of the nanostructure because of the difference in the field distribution. Thus, the strong asymmetry of HE and thermal effects in the different LCN regions is a new type of optical chiral effect.

## 3 Results and discussion

**Figure 2:**
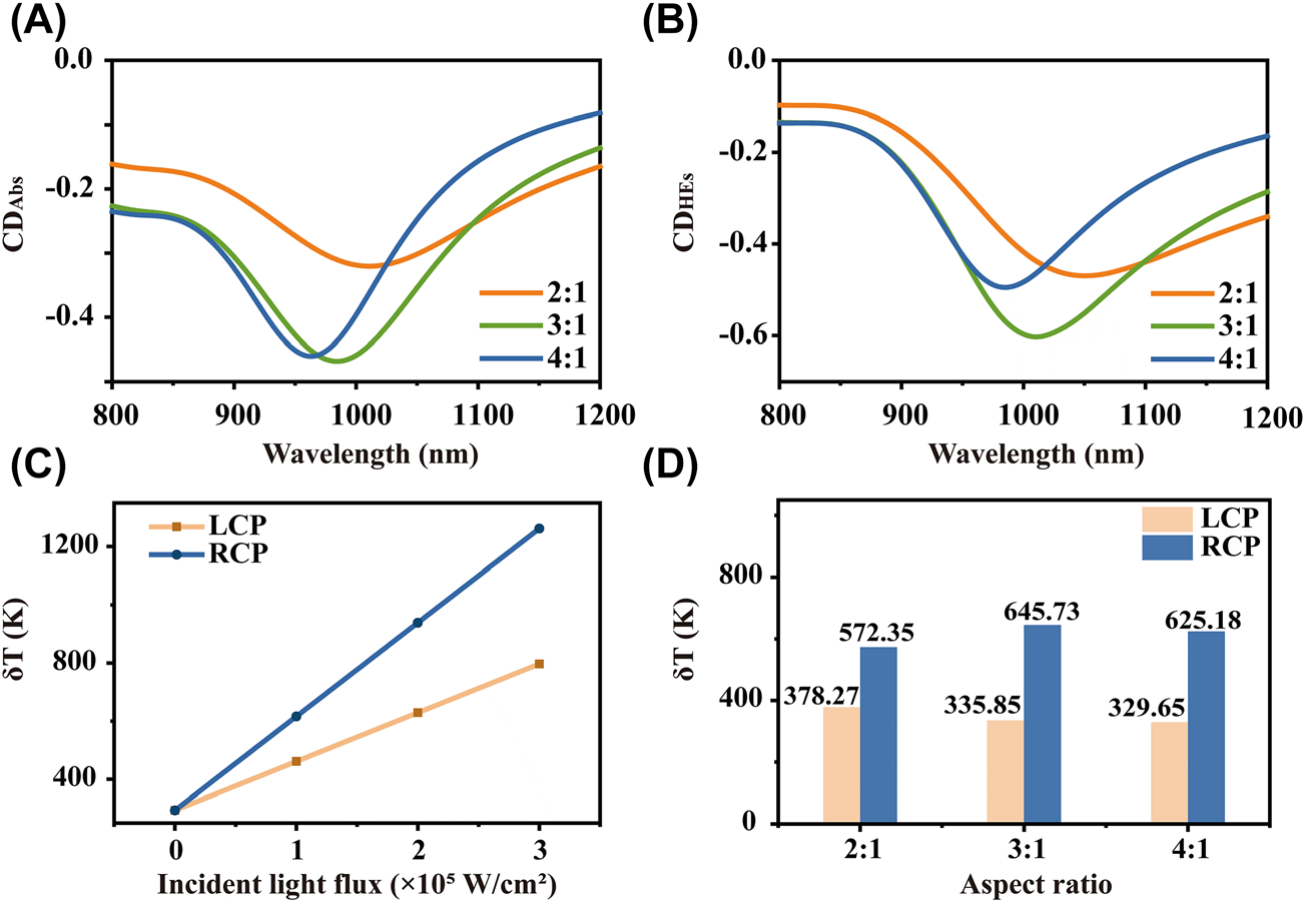
Chiral effect of LCNs with different aspect ratios. (A) Absorbance CD spectra and (B) HE generation rate CD spectra for LCNs with aspect ratios of 2:1, 3:1, and 4:1. (C) Temperature increase dependence on incident light flux in the 3:1 LCN. (D) Increase in temperature of LCNs with different aspect ratios with an incident light flux of 2 × 10^5^ W/cm^2^ and incident wavelength of 980 nm.

The chiral response of the LCN can be tuned by changing the aspect ratio between its long arm (L_1_) and short arm (L_2_), which is attributed to the breaking of different LCN symmetries. The overall length and thickness of the LCN remain 500 and 40 nm, respectively; however, the aspect ratio changes. The absorption spectra of LCNs with aspect ratios of 2:1, 3:1, and 4:1 excited by LCP and RCP are shown in Figure S3A. The strongest absorption of the LCNs appears near the wavelength of 980 nm, and its absorption ability with RCP excitation is better than that with LCP excitation. From the normalized absorption spectra (Figure S3A), CD_abs_ is calculated via Eq. (1) and the CD_abs_ spectra are shown in Figure 2A. The 3:1 LCN has the largest |CD_abs_| of ∼0.48 at the resonance wavelength, indicating the best optical chiral effect. Similarly, according to the normalized HE generation rate spectra of the LCNs (Figure S3B), the CD of the HE generation rate spectra are shown in Figure 2B. The CD_HEs_ is expressed as:

(5) $$CD_{HEs}=R_{LCP}-R_{RCP}$$

where *R*_LCP_ and *R*_RCP_ are the HE generation rates of the LCNs with LCP and RCP excitation, respectively, which are defined in Eq. (4). It can be observed that the 3:1 LCN has the largest |CD_HEs_| of ∼0.6 at the resonance wavelength.

From Figure 2C, the temperature increase is proportional to the incident light flux and the rate of temperature increase with RCP excitation is faster than that with LCP. Due to the low melting point of aluminum, a medium incident flux of 2 × 10^5^ W/cm^2^ is chosen to achieve obvious HE and thermal effects in the aluminum LCN. The temperature increase of LCNs with different aspect ratios while excited by CPL with the wavelength of 980 nm are shown in Figure 2D. The CD of temperature increases between LCP and RCP excitations (CD_T_ = *δT*_LCP_ − *δT*_RCP_) for the 2:1, 3:1, and 4:1 LCNs are −194.08, −309.88, and −295.53 K, respectively. The 3:1 LCN structure has the largest |CD_T_| of 309.88 K at the resonance wavelength. Thus, the 3:1 configuration is optimal because it has the best CD response, including optical absorption, the HE generation rate, and temperature increase (see Figure S4).

**Figure 3:**
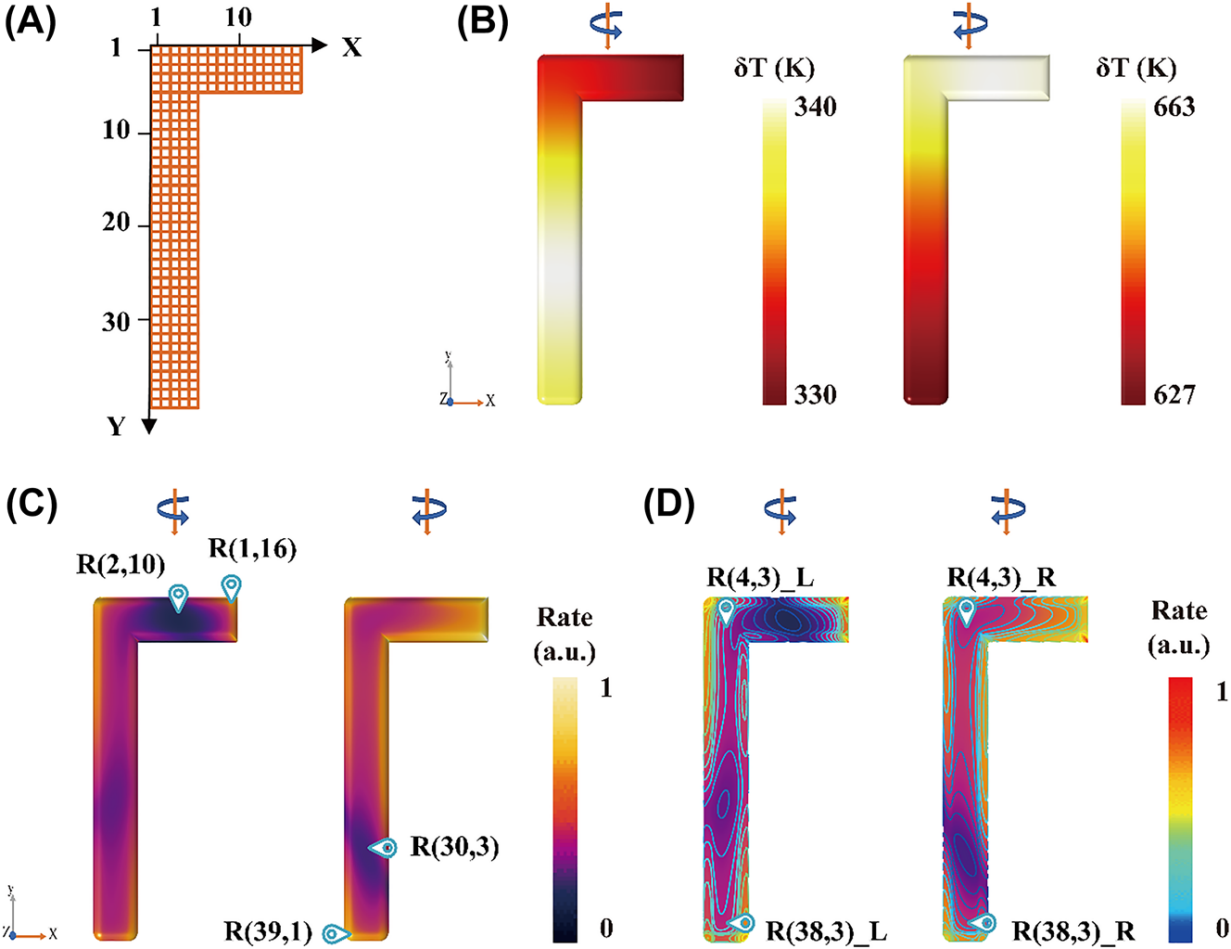
HE and thermal effects at different regions of the LCN. (A) Grid markers of the LCN surface. (B) Temperature distribution of the LCN with LCP and RCP excitations. (C) Distribution and (D) contour line indicating the HE generation rate on the LCN with LCP and RCP excitations.

The strong chiral effect achieved by using plasmonic nanostructures typically focuses on the average response of the holistic nanostructure. However, this effect is ignored across different regions of the same nanostructure. Photocatalysis performance is dependent on the region in which the molecule is adsorbed at the single-molecule level. To elaborate on the chiral effects in different regions, the upper surface of the LCN is divided into 10 × 10 nm grids for labeling, which is large enough for the tips of microcopy to detect. As shown in Figure 3A, where *R*(*x*, *y*) indicates the grid coordinate.

In Figure 3B, the temperature differences between regions of the long and short arms are only 10 and 36 K with LCP and RCP excitations, respectively. The temperature of the nanostructure after thermal equilibrium is almost the same due to the high thermal conductivity of metal. Considering that the aluminum is easily oxidized, the temperature distributions of an LCN with 3 nm Al_2_O_3_ on the its surface are shown in Figure S7A. The chiral photothermal effects of silver and gold are inconspicuous due to their higher thermal conductivity, as shown in Figures S7B and C. In practical applications, the influence of the substrate on HE and thermal effects should be considered, as shown in Figure S8. Figure 3C shows the distribution of the HE generation rate on the LCN. Aggregations of HEs are mainly focused on the region with a small curvature radius [35]. It needs to be emphasized that the HE effect is determined by the distribution of the local electric field (see Figure S5). In plasmonic photocatalysis, HE-driven chemical conversion can be synergistic with thermal excitation. However, it is difficult to distinguish the contribution of those effects in a plasmonic catalytic reaction. Although it is widely accepted that the main role in plasmonic catalysis is played by the excitation of HEs rather than the thermal effect, there have been different conclusions on this issue [40], [41], [42]. Thus, two groups of different regions with almost identical temperature but a large difference in the HE generation rate are shown in Figure 3C. The specific location of these regions is shown in Figure S2.

Group 1 [*R*(2,10) and *R*(1,16)] regions are located at the short arm of the LCN with LCP excitation and group 2 [*R*(30,3) and *R*(39,1)] regions are at the long arm of the LCN with RCP excitation. Table 1 exhibits the differences in the HE generation rate and temperature between the two regions of each group, described as a percentage of the overall surface mean. The temperature of group 1 is almost the same, with a difference of 0.41%; however, the HE generation rate shows a difference of −12.75%. The same procedure is applied for the two other regions (group 2) of the nanostructure with RCP excitation, leading to virtually identical results (for details see Table 1). The contour map of the HE generation rate helps select regions that have the same HE effect, as shown in Figure 3D. Due to the high thermal conductivity of metal, we can consider the temperatures to be consistent on the same structure. The overall average temperature of the LCN with RCP is much higher than that with LCP due to differences in absorption. *R*(4,3)_L and *R*(4,3)_R are the same region of the LCN with excitation light of different circular polarization, i.e., left and right, respectively, and the corresponding results are shown in Table 2. It can be concluded that the HE generation rate is almost the same for *R*(4,3)_L and *R*(4,3)_R; however, the temperature increase shows a large difference of −65.67% with different CPL excitation. The virtually identical results exist in *R*(38,3) with different CPL excitations (for details see Table 2). Hence, we conclude that the probed regions and the chirality of the excitation light can be adequately selected to control the HE and thermal effects of a system. It is beneficial to discuss the contribution of the HE and thermal effects in plasmon-mediated chemical reactions and to offer methods for highly efficient and selective chemical synthesis and energy conversion.

**Table 1:** HE generation rate and temperature increase of groups 1 and 2.

Varying probed region	*R*(2,10) − *R*(1,16)	*R*(39,1) − *R*(30,3)
Difference in HEs (100%)	−12.75%	−12.30%
Difference in *δT* (100%)	0.41%	0.65%

**Table 2:** HE generation rate and temperature increase of *R*(4,3) and *R*(38,3).

Varying excited light	*R*(4,3)_L − *R*(4,3)_R	*R*(38,3)_L − *R*(38,3)_R
Difference in HEs (100%)	−0.07%	−0.66%
Difference in *δT* (100%)	−65.67%	−60.10%

**Figure 4:**
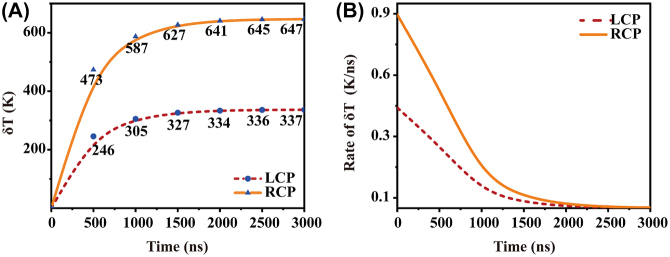
Dynamics of average temperature increases of the LCN with time evolution. (A) Average temperature increase and (B) rate of temperature increase of the LCN with LCP and RCP excitations.

In addition, the dynamics of the average temperature increase of the LCN with time evolution while excited by LCP and RCP light is shown in Figure 4. Average temperatures of the LCN increase rapidly, in less than 1000 ns, with LCP (dotted line) and RCP (solid line) excitations, and the steady-state temperature difference between them is ∼300 K. The rate of temperature change shown in Figure 4B demonstrates that changes in the temperature gradually level off and reach thermal equilibrium at ∼1500 ns. The chiral photothermal effect can be found in the temperature dynamics, which may provide potential application in the study of time-dependent catalytic processes.

Not every structure with optically induced temperature gradients and hot spots can achieve the same effects observed herein. To support this claim, the HE and thermal effects of nonstrictly chiral structures with LCP and RCP excitations are shown in Figure S10. Although the HE effect at different regions is also significantly different, there is no difference in the temperature of nonstrictly chiral structures under LCP and RCP excitations.

**Figure 5:**
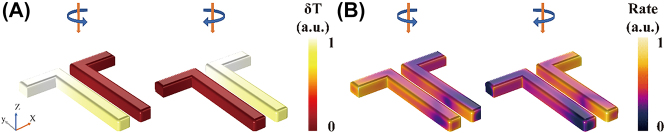
HE and thermal effects of the double-LCN. (A) Distributions of normalized temperature increase and (B) the HE generation rate of a double-LCN with LCP and RCP excitations.

Moreover, HE rate contours for depositing adsorbed molecules on nanostructures could be potentially tricky to identify in real applications. An aluminum double-LCN is constructed, as shown in Figure 5, where the LCNs on the left and right sides are defined as left-handed (LH) and right-handed (RH) chiral nanostructures, respectively. The size parameters of each LCN are consistent with those mentioned above, wherein the space between the two mirror structures is 50 nm. The average temperature and HE generation rate of the LH chiral nanostructure with LCP excitation are almost the same as those in the RH chiral nanostructure with RCP excitation. For larger molecules (such as proteins) or chemical reactions with low accuracy requirements, the double-LCN provides a stronger signal and has low molecule position requirements due to the total area of the double-LCN being larger than one region on a single LCN, which is easier to control. Thus, the double-LCN is a more suitable support for realistic reactions because it can provide a more controllable and less region-dependent strong plasmonic chiral effect.

## 4 Conclusions

In summary, the HE generation rate and temperature increase of several selected regions on a single LCN are simulated. The HE and thermal effects of aluminum L-shaped nanostructures can be locally controlled by changing the chirality of the excitation light. A significant difference in the HE effect but an almost identical thermal effect in different regions of one nanostructure can be realized by appropriately selecting the probed region. Moreover, a unanimous HE effect but a thermal effect difference of ∼60% in one region of the nanostructure can be realized by switching the polarization state of the CPL excitation. In addition, we also study the HE and thermal chiral selective effects of a double-LCN, which can be more easily controlled in terms of asymmetric chiral growth and crystallization. This work aids in the control of HE and thermal effects in high-precision photochemical reactions, as well as providing theoretical support for the design of enantioselective catalysts and an understanding of the roles of these two mechanisms in the catalytic process.

## Supplementary Material

Supplementary Material
