# Supplementary material for: Local controllability of hot electron and thermal effects enabled by chiral plasmonic nanostructures
Source: Nanophotonics. 2022 Feb 16;11(6):1195–202. doi: 10.1515/nanoph-2021-0780 (PMC11501774; doi:10.1515/nanoph-2021-0780)
Supplement: Supplementary file 1 — Supplementary Material [file j_nanoph-2021-0780_suppl.docx]

Supporting Information

**Local Controllability of Hot Electron and Thermal Effects Enabled by Chiral Plasmonic Nanostructures**

Bowen Kang,^1^ Tingting Zhang,^1^ Lei Yan,^1, *^ Chengxiang Gou,^1^ Zihe Jiang,^1^ Min Ji,^1^ Li Chen,^2, *^ Zhenglong Zhang,^1, *^ Hairong Zheng^1^, Hongxing Xu^3^

1 School of Physics and Information Technology, Shaanxi Normal University, Xi’an 710062, China

2 Department of Electrical and Computer Engineering, University of California San Diego, La Jolla, California, USA

3 School of Physics and Technology, Wuhan University, Wuhan, Hubei, China

E-mail: yanlei@snnu.edu.cn; lic095@eng.ucsd.edu; zlzhang@snnu.edu.cn

**1 Simulation**

The simulation is taken by the finite element method with COMSOL Multiphysics. The simulation model of LCN is shown in Figure S1(A). Details of the LCN array are shown in Figure S1(B), and the width (*d*) and fillet radius (*r*) are 50 and 10 nm, respectively. Optical and thermal calculations are carried out by two modules, “Electromagnetic Waves, Frequency Domain (ewfd)” and “Heat Transfer in Solids (ht)”, respectively. The calculations are performed in a unit cell of the array with periodic boundary conditions applied to the side boundaries. The y-z cross sectional view of the chiral system is shown in Figure S1(C). The LCP and RCP beams perpendicularly illuminate the nanostructure from the top boundary. The reflected light is collected at the top boundary through the scattering matrix element. To calculate the distributions of the temperature increases, the boundary conditions in the heat transfer equation are set as T_0_ = 293.15 K for both the uppermost and nethermost boundaries. Under continuous wave excitation of a plasmonic structure, the local temperature generated by LSPR will increase. Using metallic nanoparticles as nanosources of heat, the heat power intensity is determined by the $Q(\boldsymbol{r})=\sigma I_{0}$. The absorption cross section is obtained by the integration of ohmic heating within the whole nanostructure. Thus, we select the “electromagnetic loss” of LCN in the “ewfd” field at the heat source in the solid heat transfer field, which can couple the two fields. Based on the resolution of the heat diffusion equation, heat is transferred from the LCN to the external medium, and the temperature gradually reaches equilibrium after excitation.


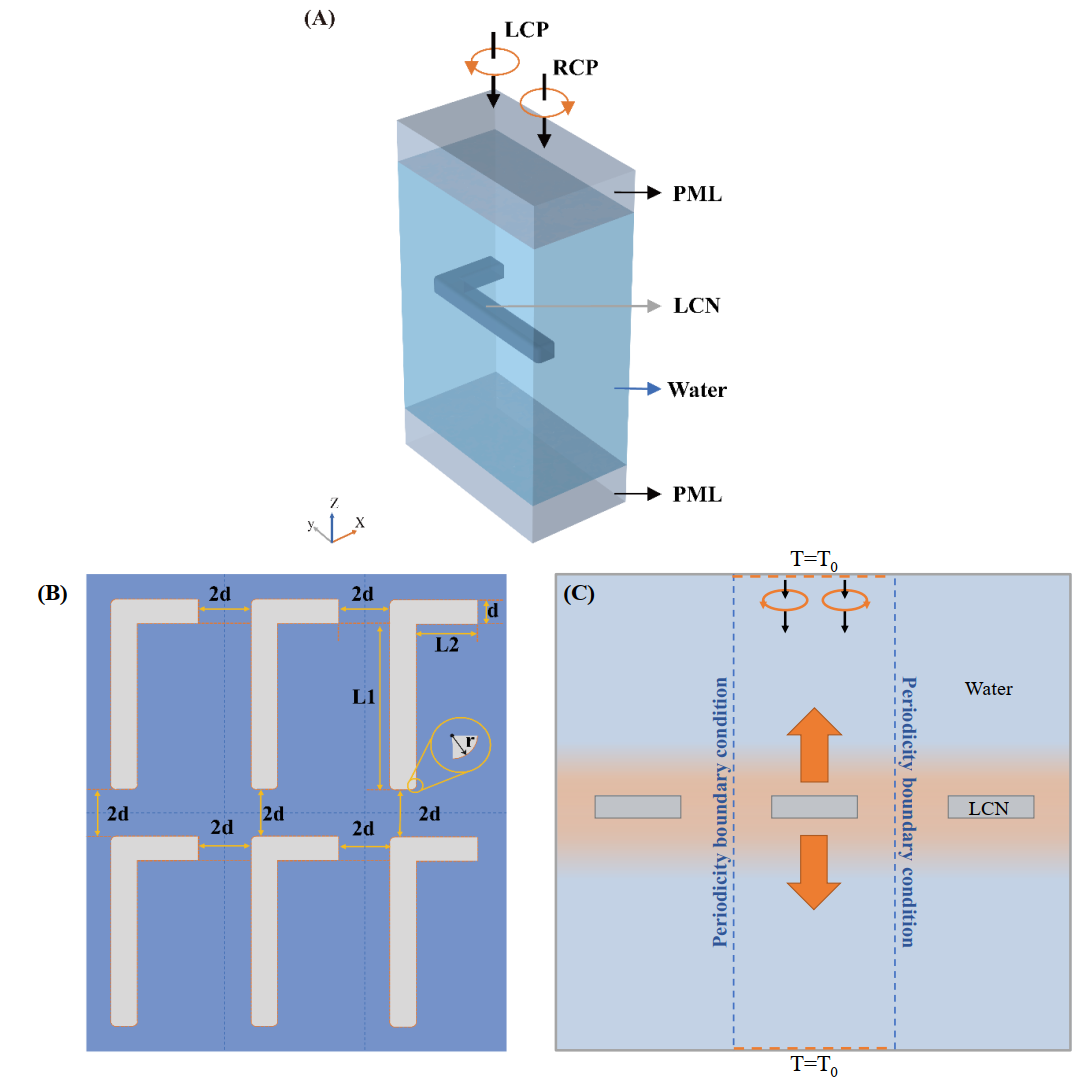


**Figure S1.** (A) Simulation model of LCN. The material of LCN is aluminum and its refractive index is from McPeak et al [1]. The blue part is water. (B) The x-y cross sectional view of the LCN periodic array. The periodic arrays are spaced every 2d (100 nm). The length of the long arm and short arm is L_1_ and L_2_, respectively. (C) The y-z cross sectional view of the chiral system.


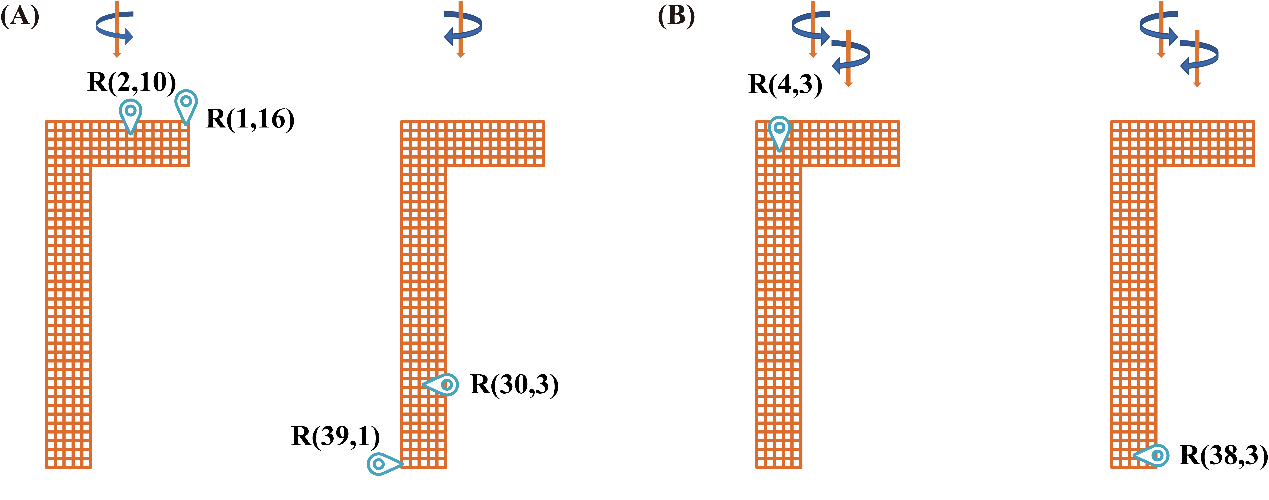


**Figure S2.** (A) Grid markers of group 1 and group 2. (B) Grid markers of R (4,3) and R (38,3).

**2 Optical chiral effect of LCNs**

The localized surface plasmon resonance (LSPR) of metal nanostructures can be simply derived from a metal sphere that is much smaller than the illumination wavelength. In this case, the polarizability is as follows:

$\alpha(\omega)=4\pi R^{3}\frac{\varepsilon(\omega)-\varepsilon_{S}}{\varepsilon(\omega)+2\varepsilon_{S}}$ (1)

where *ε(ω)* is the permittivity of the metal sphere, *ε_s_* is the permittivity of the external medium, and *R* is the radius of the sphere. The absorption cross section is written as [2]:

$\sigma=kIm (\alpha)-\frac{k^{4}}{6\pi}\left| \alpha\right|^{2}$ (2)

where $k=2\pi/\lambda$. Optical property of the LCN is simulated with COMSOL Multiphysics through finite element method. Absorption cross section is obtained by the integration of ohmic heating within the whole nanostructure, which can be expressed as

$\sigma=\frac{1}{I}\iiint Q_{rh} dV$ (3)

where *Q_rh_* is the power loss density in the simulated nanostructure, *I* is the incident intensity of the light, and *V* is the volume of the nanostructure. According to Equation 4 in the main text, the rate of HEs generation from plasmon decay is proportional to the square of the electric field intensity, hence the value of *log |E_normal_|^2^* can be used for evaluating the rate of HEs generation.


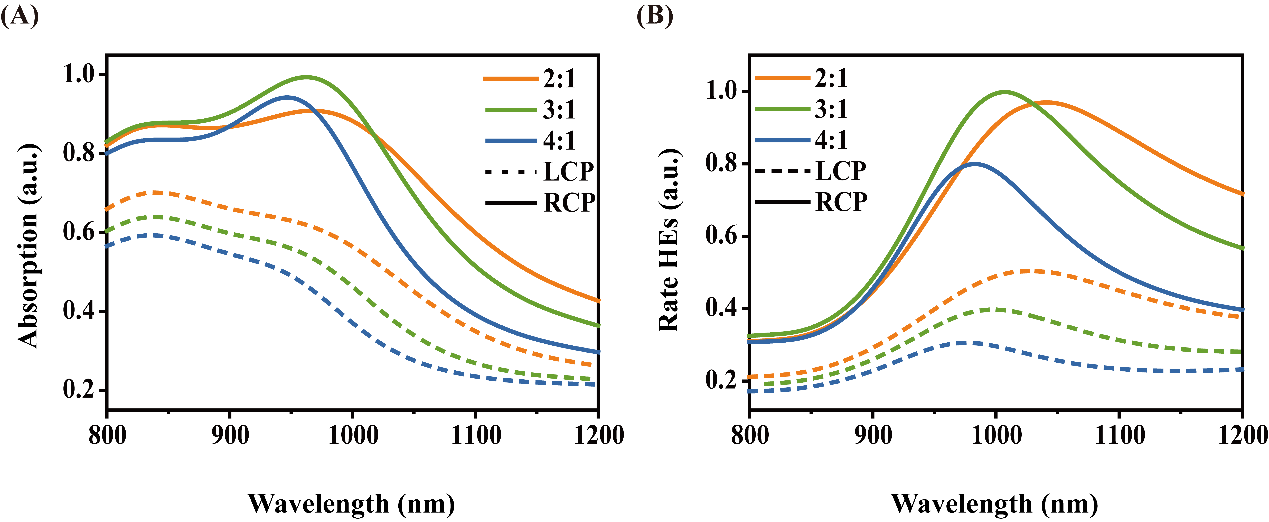


**Figure S3.** (A) Absorbance spectra and (B) hot electrons generation rate spectra of LCNs with different ratios. This ratio is defined as the length of the long arm divided by the length of the short arm.

The circular dichroism of absorption, the circular dichroism of HE effect, and differences in average temperature increase with LCP and RCP excitation of the 2:1, 3:1, and 4:1 structures are compared. It can be found that the structure with a ratio of 3:1 has the largest absorption circular dichroism, HE effect circular dichroism, and the difference in average temperature increase. Specifically, its optical chirality and photothermal chirality are both the strongest. Hence, the 3:1 configuration is the optimal one.


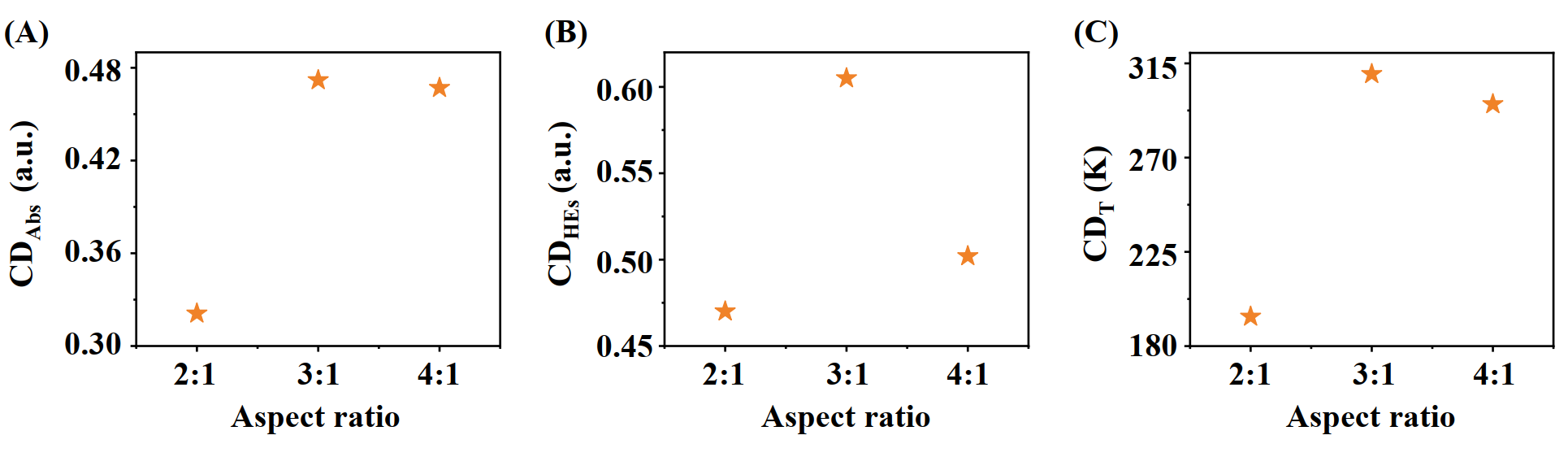


**Figure S4.** (A) CD_abs_, (B) CD_HEs_ and (C) CD_T_ depend on the aspect ratio curve.


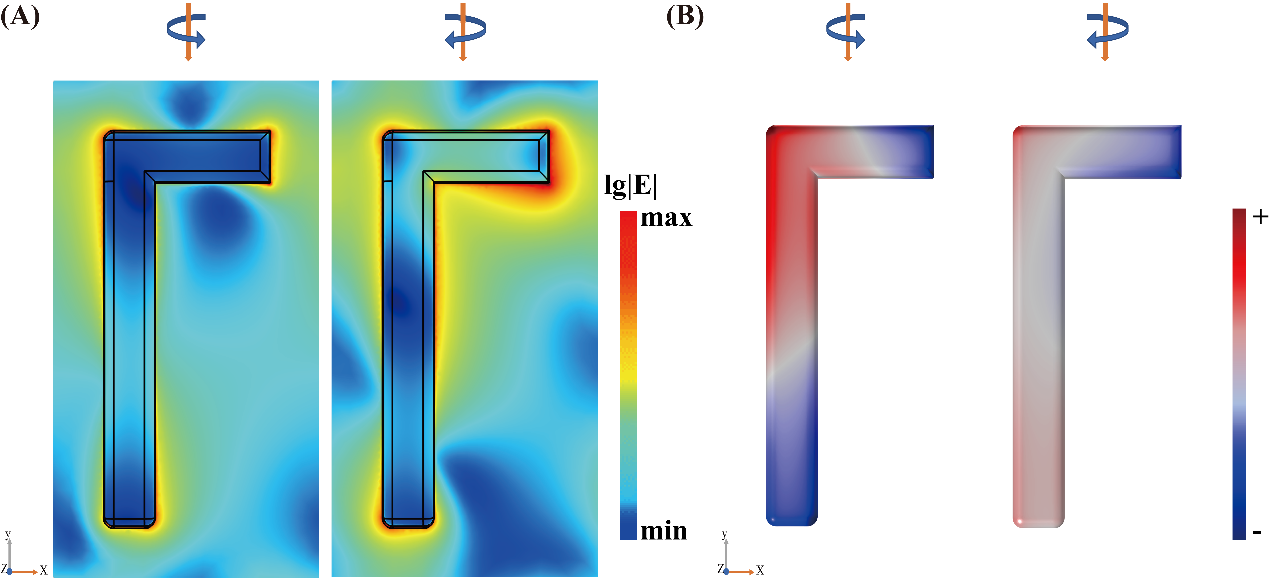


**Figure S5.** (A) Distribution of electric field in x-y plane of 3:1 LCN with LCP and RCP excitation, respectively. (B) Charge distribution in the surface of 3:1 LCN with LCP and RCP excitation, respectively.

**3 Chiral thermal effect of LCNs**

As shown in Figure S6, the different nanostructures have great differences in temperature increases between LCP and RCP excitation, and the largest temperature difference occurs in the 3:1 structure used in the main text. Figure S7(a) shows that the average temperature increases of LCN with Al_2_O_3_ excited by LCP are greater than those of LCN in the main text, and the opposite is true for RCP. Thus, the temperature difference becomes smaller and the chiral effect becomes worse of LCN with Al_2_O_3_. In Figures S7(b) and (c), the temperature difference between the long and short arms is small since the thermal conductivities of both silver and gold are higher than those of aluminum, and the photothermal chiral effect is also worse. By changing the external medium or increasing the incident light flux, the photothermal chiral effect can be enhanced.


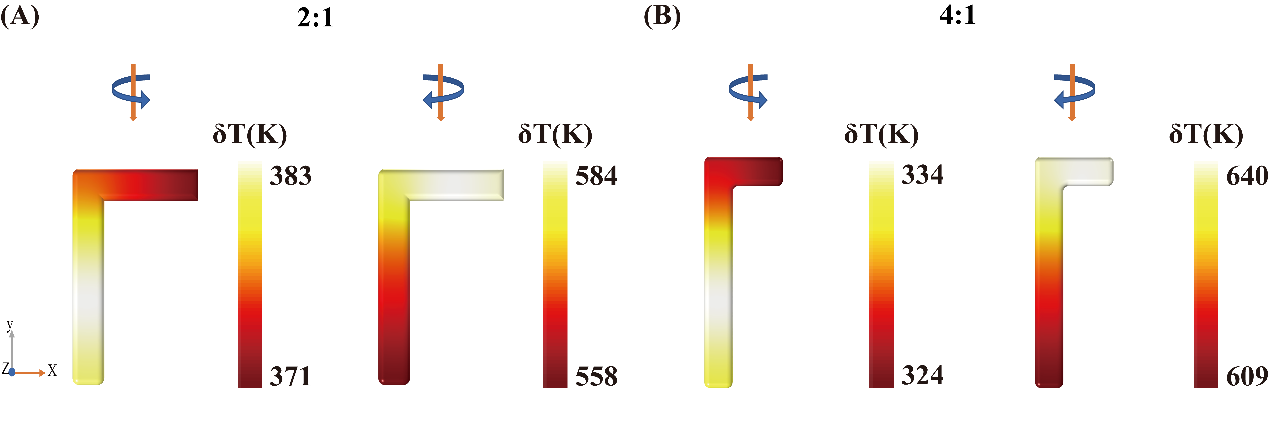


**Figure S6.** Distribution of temperature increases on the LCNs with (A) 2:1 ratio and (B) 4:1 ratio.


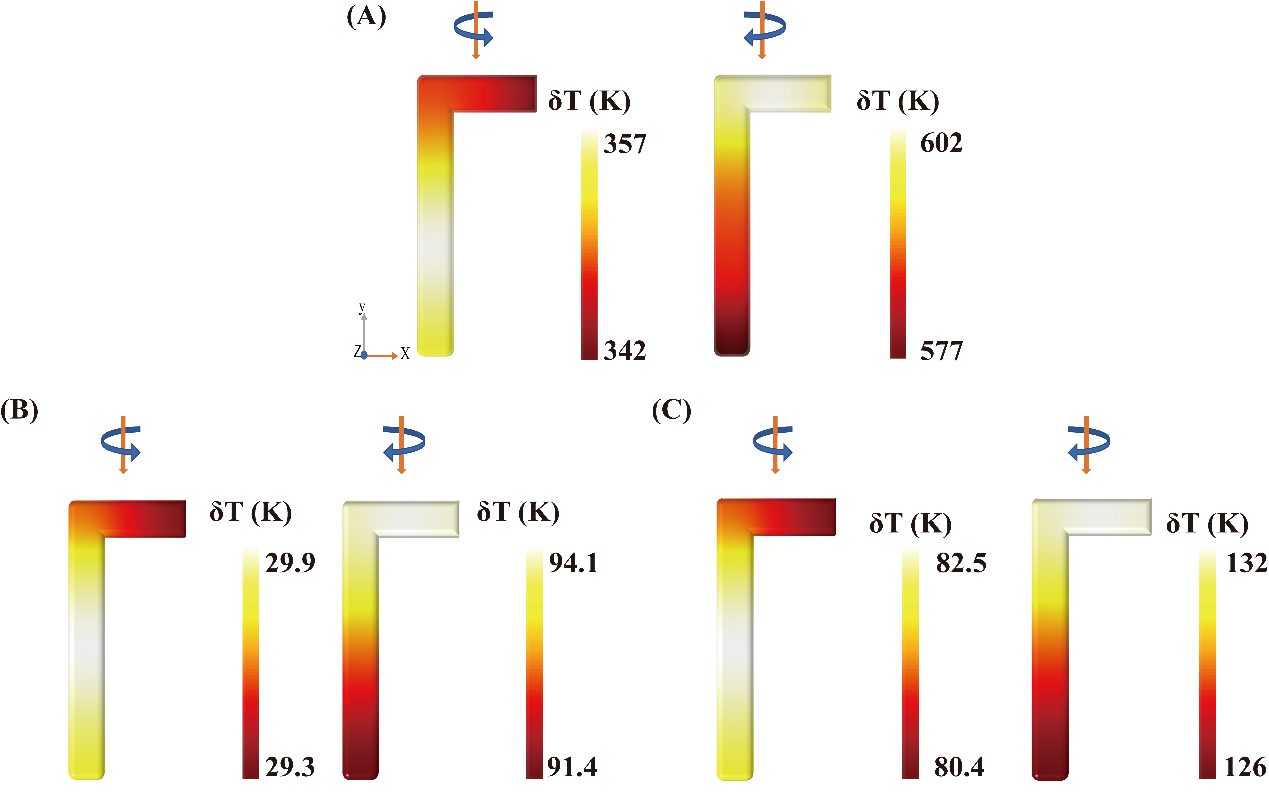


**Figure S7.** (A) Distribution of temperature increases of the aluminum LCN with 3 nm Al_2_O_3_ on the surface. Distributions of temperature increase of (B) silver and (C) gold LCNs.

Periodicity boundary conditions and initial temperature set in COMSOL are consistent as those in above. The substrate is set as glass, which has a low thermal conductivity (see Figure S8A). The thermal conductivity of glass (1.4) is greater than that of water (0.6). Most of the heat produced in LCN is transmitted to the substrate, as shown in Figure S8B.


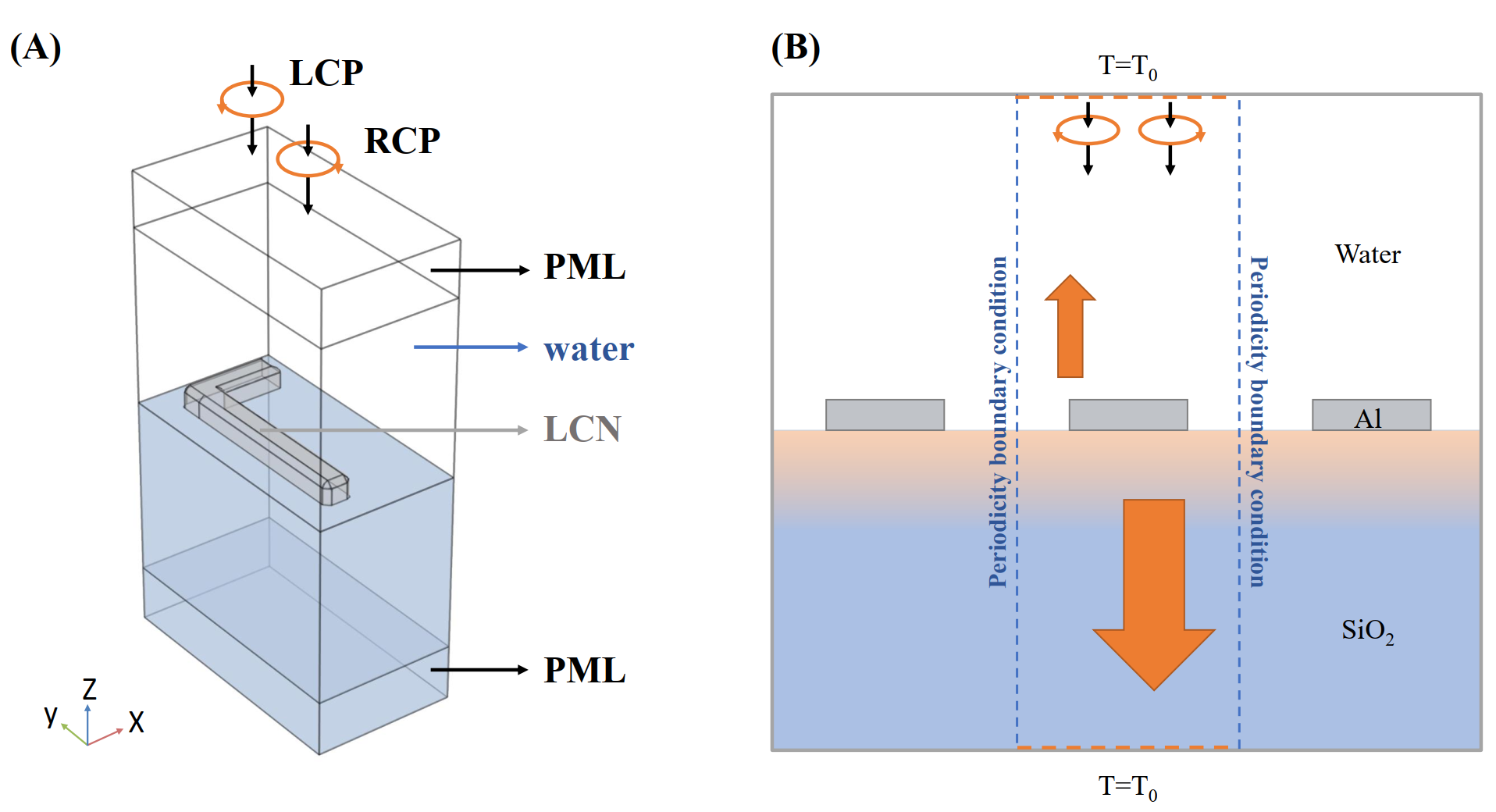
 **Figure S8.** Simulation model of LCN on the substrate. (A) Simulation model of LCN on the substrate. (B) The y-z cross sectional view of the chiral system on the substrate.

Similarly, distributions of the HE generation rate and temperature increase of the LCN on the substrate are simulated and shown in Figure S9. The thermal chiral effects of the LCN are weakened but still exist with LCP and RCP excitation. And the hot electron effect of LCN is nearly identical with or without a substrate (Figure S9(B)). That is to say, the chiral effects of LCN as the description in the manuscript still exist and can be the reference to the real experiment. It should be emphasized that, materials with lower thermal conductivity can be selected in practical experiments to reduce the influence of the substrate on structural chiral thermal effect. In the main text, we just discussed the single structure, which benefits the mechanisms of distinguishing between HE and thermal effects via the simple system.


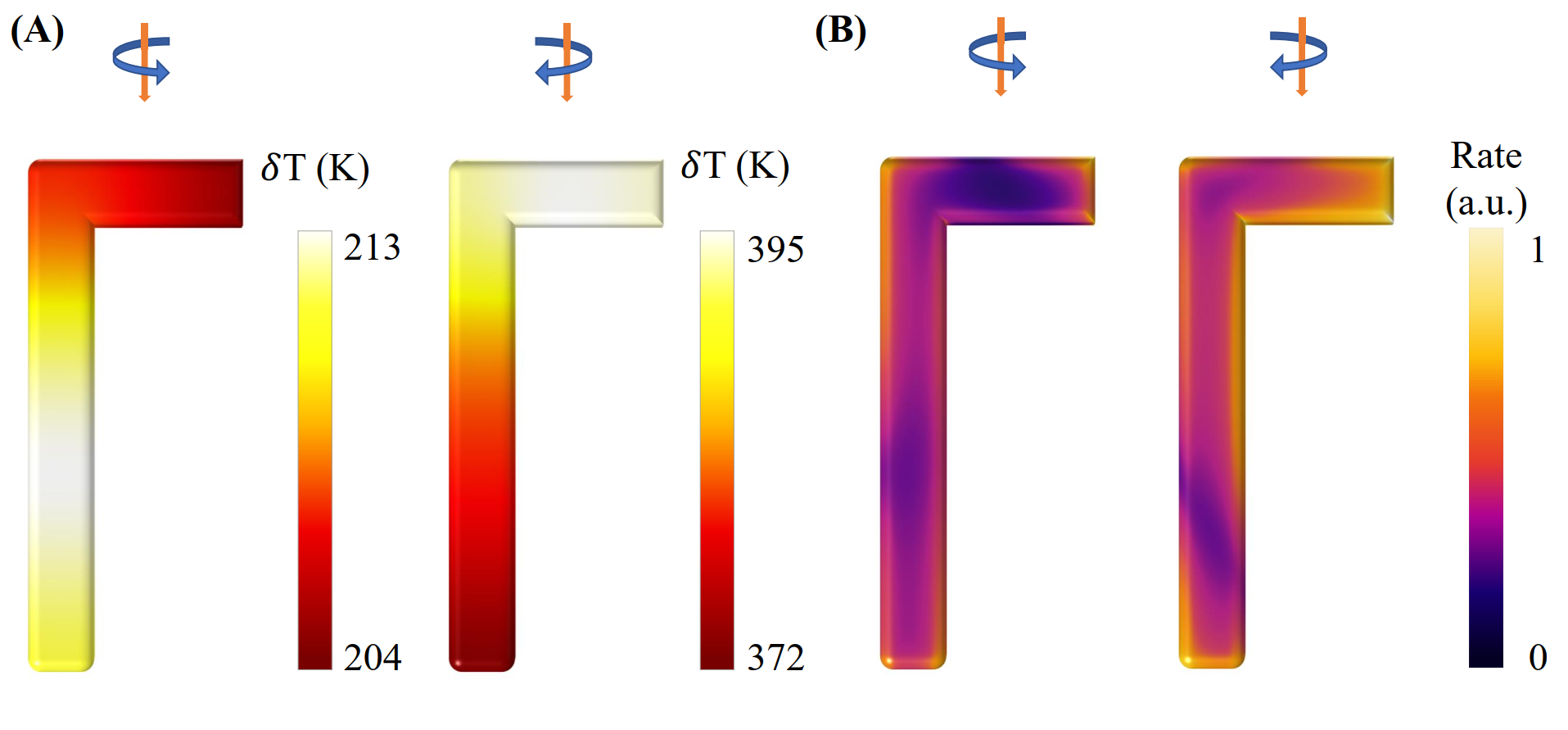
 **Figure S9.** (A) Temperature distribution of the LCN on the substrate with LCP and RCP excitation. (B) HE generation rate distribution of the LCN on the substrate with LCP and RCP excitation.


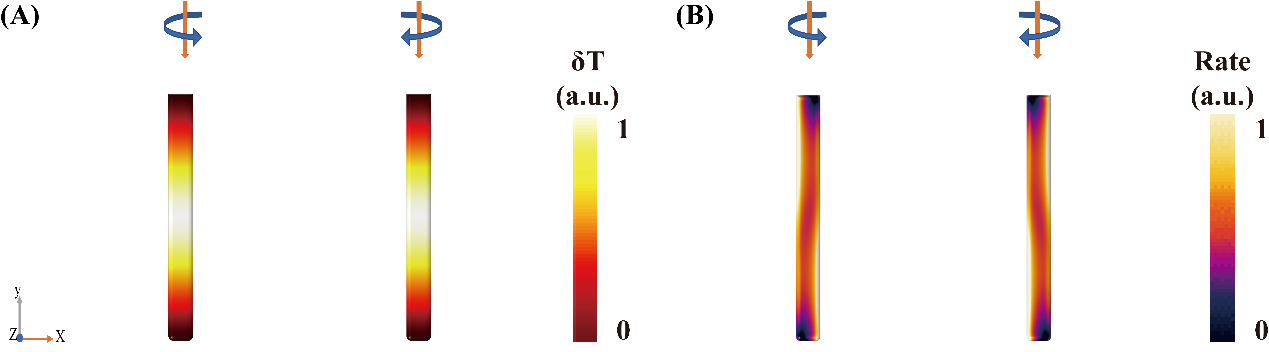


**Figure S10.** (A) Distributions of normalized temperature increase and (B) HEs generation rate of non-strictly chiral structure with LCP and RCP excitation.

References

[1] K. M. McPeak, S. V. Jayanti, S. J. Kress, S. Meyer, S. Iotti, A. Rossinelli, and D. J. Norris, "Plasmonic Films Can Easily Be Better: Rules and Recipes," ACS Photonics 2, 326-333 (2015).

[2] G. Baffou and R. Quidant, "Thermo-plasmonics: using metallic nanostructures as nano-sources of heat," Laser & Photonics Reviews 7, 171-187 (2013).
